# Data modeling positive security behavior implementation among smart device users in Indonesia: A partial least squares structural equation modeling approach (PLS-SEM)

**DOI:** 10.1016/j.dib.2020.105588

**Published:** 2020-04-22

**Authors:** Kautsarina, Achmad Nizar Hidayanto, Bayu Anggorojati, Zaenal Abidin, Kongkiti Phusavat

**Affiliations:** a Faculty of Computer Science, Universitas Indonesia, Indonesia; b Ministry of Communication and Informatics, Indonesia; c Universitas Negeri Semarang, Indonesia; d Kasetsart University, Thailand

**Keywords:** End-user, positive security behavior, smart devices, structural equation modeling, Indonesia

## Abstract

The article presents raw inferential statistical data related to understanding the positive security behaviors of smart device users in Indonesia, which was used to determine whether the studied variables were direct or mediating factors. The factors explored include government efforts, technology provider support, privacy concerns, trust, perceived behavioral control, attitudes, and subjective norms. The theory of planned behavior was adopted to develop the proposed model for implementing positive security behaviors. Structured questionnaires were distributed via an online survey to consumers currently using a smartphone or using a smartphone and some other smart device. Furthermore, the respondents were from 19 provinces in Indonesia. The quantitative research method was used to analyze the data. Reliability and validity were confirmed. Structural equation modeling (SEM) using the Smart PLS software version 3 was used to present data. SEM path analysis identified estimates of the relationships of the primary constructs in the data. The outcomes obtained from this dataset demonstrate a direct influence between government efforts, privacy, and perceived behavioral control and performing positive security behaviors. Other variables had positive and significant influences on implementing positive security behaviors, indicating their roles as mediation variables. This data is useful for reference and consideration in the improvement of smart device users’ security behaviors. This data can also provide valuable insights to countries with characteristics that are similar to those of Indonesia.

Specifications Table

**Table:** 

Subject	Computer science (general)
Specific subject area	Information system
Type of data	Table
	Chart
	Figure
How data were acquired	The researchers developed a questionnaire that included demographic data and research questions related to the variables being investigated, which were factors, such as government efforts, technology provider support, trust, and privacy, as well as attitudes, subjective norms, and perceived behavioral control. The data was acquired by distributing the questionnaire as an online survey to individuals who use a smartphone and individuals who use a smartphone and some other smart device in some regions in Indonesia.
Data format	Raw
	Analyzed
	Descriptive and Statistical Data
Parameters for data collection	The sample consisted of a smartphone user and user of the smartphone and another smart device (s). The questionnaire was distributed as an online survey to users in several regions in Indonesia.
Description of data collection	The researchers disseminated the survey link to the online communication channel using WhatsApp. Recipients who were willing to participate in the study filled out the online survey. The original questionnaire in Bahasa is provided in link: s.id/privasiperangkatpintar. The questionnaire in English is provided as a Supplementary File.
Data source location	Respondent Locations: 19 provinces (in alphabetical order): Bali, Bangka Belitung, Banten, Bengkulu, Yogyakarta, Jakarta, Jambi, West Java, Central Java, East Java, East Kalimantan, Lampung, North Maluku, Central Sulawesi. North Sulawesi, Southeast Sulawesi, West Sumatera, South Sumatera, and North Sumatera Country: Indonesia
Data accessibility	Repository name: Mendeley Data
	Data identification number: 10.17632/tnf63kt4jf.2
	Direct URL to data:
	https://data.mendeley.com/datasets/tnf63kt4jf/draft?a=6da985d2-e311-4a85-9002-677121795259

## Value of the Data

• The data is useful for all stakeholders, such as technology providers, academicians, especially the government of Indonesia, in terms of improving security awareness efforts among smart device users.
• The data presents how government efforts, technology provider support, trust, privacy concerns, attitudes, subjective norms, and perceived behavioral control impact smart device users’ positive security behaviors. This information is useful because it can serve as a reference and be considered in the development of measures to improve smart device users’ security behaviors.
• This data can be used to develop a measurement tool to determine the positive security behaviors related to the use of smart devices in another context.
• This data can provide useful insights for countries with characteristics that are like those of Indonesia.

## 1 Data Description

The facts and statistics presented in this paper were collected via primary data collection through an online survey, which can be accessed at the following link: s.id/privasiperangkatpintar (in Bahasa). The questionnaire in English is provided as a Supplementary File. The researchers developed the survey instrument using research constructs based on previous studies, as shown in Table 1.

**Table 1:** Research variables of the survey.

Variable	Indicator	Reference
Stakeholder involvement		
- Government efforts	1. Existing regulations protect against the misuse of personal information.	[1,2]
	2. Existing regulations govern how personal information is collected and used.	
	3. Regulations control the use of sanctions for violations or misuse of personal data.	
	4. The government has provided training to increase security awareness.	
	5. The existing program has educated users about the responsibilities of smart device users.	
	6. The existing program has educated users about the consequences of using smart devices.	
- Technology provider support	1. The privacy policy statement is clear and understandable.	[3,4]
	2. Existing privacy policies make me more aware of my rights.	
	3. Providers use reliable technology to protect my privacy.	
	4. Providers give flexibility for me as the user to manage the mechanism for securing my data.	
User concerns		
- Privacy concerns	1. I feel disturbed when the provider asks for personal information.	[5], [6], [7]
	2. I think about considering privacy before giving personal data.	
	3. I object to providing personal data.	
	4. Providers collect too much of my personal information.	
	5. Providers should work harder to secure users’ personal information.	
- Trust in technology	1. I feel comfortable that the provider protects the data well.	[1]
	2. I can count on the provider not to misuse users’ permissions.	
	3. I can depend on the provider to comply with all government regulations related to protecting user data.	
Theory of Planned Behavior		
- Perceived behavioral control	1. I have control over the personal information released by smart devices.	[8]
	2. I have control over anyone who can gain access to personal information.	
	3. I have control over how device providers use my personal information.	
	4. I am sure I can control my personal information.	
- Attitudes	1. Applying security measures to smart devices is a good thing.	[8]
	2. Taking security measures on smart devices is important.	
- Subjective norms	1. Esteemed colleagues believe that I must maintain my personal information.	[8]
	2. My family believes that I must be careful about exposing my personal information.	
	3. Influential community leaders believe that I must be careful about exposing my personal information.	
- Positive security behavior	1. Reading the privacy policy statement carefully before using the device is important.	[1]
	2. I know where to report an incident related to smart devices' security.	
	3. I know of privacy issues related to the use of smart devices that I have.	
	4. I know how to control the personal information given to smart devices.	
	5. I can control the protection of my personal information on all smart devices that I have.	

The wording of the questionnaire was initially developed in English and then translated into the local language (Bahasa). The survey was divided into two parts. Part A addressed demographic information, including respondents’ age, gender, educational qualifications, and smart device ownership. Part B included questions covering the different constructs in the proposed research model using a five-point Likert scale ranging from (1) “strongly disagree” to (5) “strongly agree.”

The online communication channel, namely WhatsApp, was used to distribute the questionnaire. After eliminating invalid responses, that is, 18 respondents filled incomplete questionnaires; data from 314 respondents were analyzed. The demographic characteristics of the respondents are shown in Table 2.

**Table 2:** Demographic characteristics (N = 314).

Measure	Item	Count	%
Gender	Male	148	47.1
	Female	165	52.5
	Prefer not answered	1	0.4
Age	<20	10	3
	21–30	96	31
	31–40	156	50
	41–50	21	7
	51–60	28	9
	>60	3	1
Education	High school or below	20	6.4
	Associate and bachelor's degree	143	45.5
	Master's degree or higher	151	48.1
Occupation	Student	10	3
	Employed	276	87.9
	Unemployed	18	9.1
Ownership of smart devices besides a smartphone	Yes	106	33.8

**Fig. 1:**
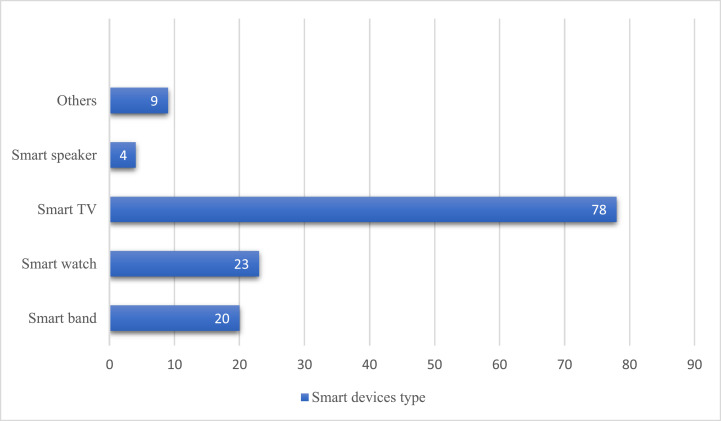
The types of smart devices owned by the respondents.

**Fig. 2:**
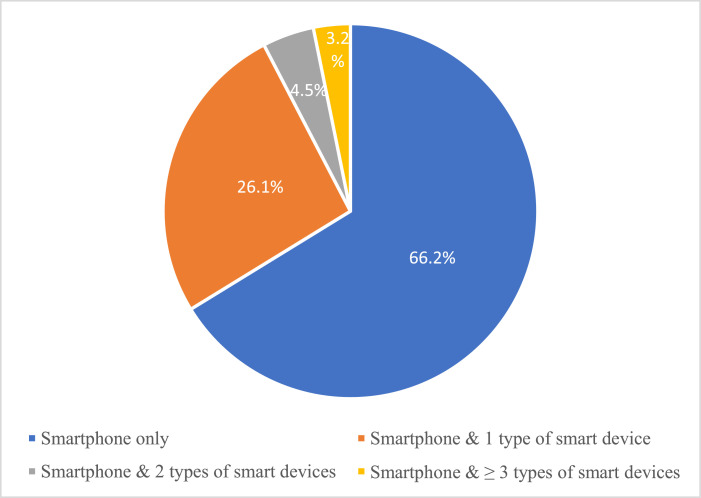
Ownership of smartphones and other types of smart devices.

The graph in Fig. 1 shows the kinds of smart devices owned by the respondents. Among the 106 respondents with a smart device besides a smartphone, 78 respondents (around 73.6%) owned a smart TV. Furthermore, the chart in Fig. 2 reveals that about 7.7% of the 106 respondents had more than one type of smart device.

## 2 Experimental Design, Materials, and Methods

The presented data were collected based on quantitative research methods. A survey method was chosen as the preferred technique because it provides many benefits, including allowing the collection of standardized data, which enabled researchers to meet the aim of the research [9,10], namely, understanding the factors that influence smart device users’ positive security behaviors.

Current smart device users and smartphone users, who were assumed to be potential adopters of other smart devices in some regions in Indonesia, were selected as respondents. The researchers proposed a model to test the data. The model consists of constructs: government efforts, technology provider support, trust, and privacy, as well as attitudes, subjective norms, and perceived behavioral control, could directly influence positive security behavior or serve as mediation variables to influence positive security behavior. The quality of the measurement model was determined based on its validity and reliability by considering the following values: Cronbach's alpha (> 0.60), composite reliability (>0.70), average variance extracted (AVE)(> 0.50), and loading factor (0.70) [11]. The measurement accuracy data can be seen in Table 3.

**Table 3:** Measurement Model.

construct Research	PLS code item	Cronbach's alpha	Composite reliability	Average variance extracted (AVE)	Factor loadings	P-values
Government efforts (GE)	GE1	0.879	0.908	0.622	0.770	0.000
	GE2				0.824	0.000
	GE3				0.847	0.000
	GE4				0.765	0.000
	GE5				0.770	0.000
	GE6				0.750	0.000
Technology provider support (TS)	TS1	0.835	0.890	0.669	0.818	0.000
	TS2				0.833	0.000
	TS3				0.838	0.000
	TS4				0.781	0.000
Trust (TT)	TT1	0.899	0.936	0.831	0.864	0.000
	TT2				0.951	0.000
	TT3				0.917	0.000
Privacy (PC)	PC1	0.835	0.882	0.600	0.710	0.000
	PC2				0.808	0.000
	PC3				0.798	0.000
	PC4				0.821	0.000
	PC5				0.731	0.000
Attitudes (ATT)	ATT1	0.871	0.939	0.886	0.947	0.000
	ATT2				0.936	0.000
Subjective norms (SN)	SN1	0.797	0.880	0.710	0.861	0.000
	SN2				0.894	0.000
	SN3				0.768	0.000
Perceived behavioral control (PBC)	PBC1	0.929	0.950	0.825	0.890	0.000
	PBC2				0.920	0.000
	PBC3				0.929	0.000
	PBC4				0.893	0.000
Positive security behavior (PSB)	PSB2	0.860	0.905	0.704	0.775	0.000
	PSB3				0.828	0.000
	PSB4				0.886	0.000
	PSB5				0.863	0.000

**Fig. 3:**
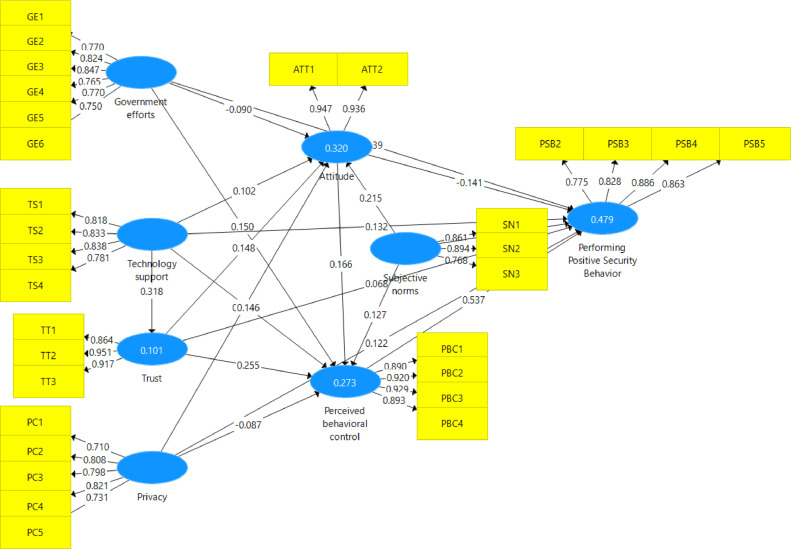
Measurement and structural model analysis.

The proposed research model was used to empirically analyze the data using the partial least squares structural equation modeling (PLS-SEM) technique, and SmartPLS version 3 software was used to code the data and run the statistical analysis. PLS-SEM is known to be reliable in sample distribution and small sample size. The structural model can be seen in Fig. 3.

The structural model was examined by testing the hypothesized relationships. Moreover, the bootstrapping method was used on 5,000 subsamples to assess the significance and path coefficients, as suggested by Hair et al. [12]. The output model analysis data is displayed in Table 4.

**Table 4:** Outcomes of structural equation modeling analysis.

Path	Hypothesis	Path Coefficient(β)	T-statistics	P-values	Supported?
Government efforts > Positive security behavior	H1 (+)	0.139	2.321	0.020	Yes
Government efforts > Attitudes	H2 (+)	-0.090	1.671	0.095	No
Government efforts > Perceived behavioral control	H3 (+)	0.151	2.483	0.004	Yes
Technology provider support > Positive security behavior	H4 (+)	0.132	1.876	0.061	No
Technology provider support > Attitudes	H5 (+)	0.102	1.698	0.090	No
Technology provider support > Perceived behavioral control	H6 (+)	0.146	2.320	0.020	Yes
Technology provider support > Trust	H7 (+)	0.318	5.300	0.000	Yes
Trust > Positive security behavior	H8 (+)	0.068	1.327	0.184	No
Trust > Attitudes	H9 (+)	0.148	3.000	0.003	Yes
Trust > Perceived behavioral control	H10 (+)	0.255	4.084	0.000	Yes
Privacy > Positive security behavior	H11 (+)	0.122	2.560	0.011	Yes
Privacy > Attitudes	H12 (+)	0.423	7.762	0.000	Yes
Privacy > Perceived behavioral control	H13 (+)	-0.087	1.588	0.112	No
Attitudes > Perceived behavioral control	H14 (+)	0.166	2.876	0.004	Yes
Subjective norms > Attitudes	H15 (+)	0.215	3.372	0.001	Yes
Subjective norms > Perceived behavioral control	H16 (+)	0.127	2.107	0.035	Yes
Attitudes > Positive security behavior	H17 (+)	-0.141	2.960	0.003	No
Subjective norms > Positive security behavior	H18 (+)	0.041	0.812	0.417	No
Perceived behavioral control > Positive security behavior	H19 (+)	0.537	11.487	0.000	Yes

### 2.1 Ethical considerations

The researchers ensured that respondents were well informed about the background and the aim of this research. Respondents were also assured of the confidentiality of the data they submitted in the survey.

### 2.2 Academic, practical, and policy implications of this data article

The data presented in this article offers implications for the academic field. Some variables directly influenced users’ performance of positive security behaviors, while other variables had positive and significant values, indicating their roles as mediation variables. For example, among the constructs of the theory of planned behavior, the data indicates that only perceived behavioral control directly influenced positive security behavior, given the strong relationship between them (β = 0.537). Meanwhile, subjective norms influenced attitudes (β = 0.215) and perceived behavioral control (β = 0.127) in a positive and significant way, and attitudes influenced perceived behavioral control with a path coefficient of β = 0.166. Therefore, among academics in the security awareness field, this finding can enhance understanding of how mediation variables can lead users actually to perform positive security behaviors.

The data also indicates that government efforts directly influenced positive security behavior in a positive and significant way, as indicated by a path coefficient of β=0.139. Based on Fig. 3, R^2^ demonstrates that the research model explains 47.9% of the variance in performing positive security behavior. Furthermore, the data indicates that government efforts influenced perceived behavioral control in a positive and significant way with a path coefficient of β = 0.151. The present findings also note there are more people use more than one smart device in addition to their smartphone. Regarding practical implications, the data presented in this article can help policymakers who are developing security policies enhance users’ positive security behaviors. Overall, insights from this dataset can be used to create new strategies and guide the revision of existing policies.

## Appendix Supplementary materials

Image, application 1

Image, application 2
