# Supplementary material for: Data modeling positive security behavior implementation among smart device users in Indonesia: A partial least squares structural equation modeling approach (PLS-SEM)
Source: Data Brief. 2020 Apr 22;30:105588. doi: 10.1016/j.dib.2020.105588 (PMC7200857; doi:10.1016/j.dib.2020.105588)
Supplement: Supplementary file 2 [file mmc2.docx]

**Smart Device User Perception towards Privacy Questionnaire**

Hello, welcome to the survey page.

I am a doctoral student from the Faculty of Computer Science, Universitas Indonesia.

Currently, I am researching ‘Smart Device User Perceptions of Privacy.’ As part of my research process, I want to ask your opinion as a smart device user regarding privacy issues. This survey takes about 5 – 10 minutes. Please readily fill out this survey. If you have questions regarding this questionnaire, don’t hesitate to contact me at email: [kautsarina61@ui.ac.id](mailto:kautsarina61@ui.ac.id).

I appreciate your help and participation.

Sincerely,

Kautsarina

Information: There are ten souvenirs for lucky respondents who will be drawn at the end of the questionnaire filling period. Be sure to fill in an email to get this opportunity.

1. **Profile of Respondents**

This section consists of information that describes your profile as a respondent. This information for research purposes only.

1. Your email address : ____________________________

(Please fill in your email address to get the chance to get a souvenir)

1. Province of Domicile *(Please tick the appropriate box)*:

- Jakarta Capital Special Region
- West Java
- Central Java
- East Java
- Banten
- Special Region of Yogyakarta
- Nanggroe Aceh Darussalam
- East Sumatera
- West Sumatera
- Riau
- Riau Island
- Jambi
- South Sumatera
- Bangka Belitung
- Bengkulu
- Lampung
- West Nusa Tenggara
- East Nusa Tenggara
- West Kalimantan
- Central Kalimantan
- South Kalimantan
- East Kalimantan
- North Kalimantan
- North Sulawesi
- West Sulawesi
- Central Sulawesi
- Southeast Sulawesi
- South Sulawesi
- Gorontalo
- Maluku
- North Maluku
- Papua
- West Papua

1. Sex *(Please tick the appropriate box)* :

- Female
- Male
- Prefer not answered

1. Range of Age *(Please tick the appropriate box)* :

- Less than or equal to 20 years old
- 21 – 30 years old
- 31 – 40 years old
- 41 – 50 years old
- 51 – 60 years old
- Above or equal to 61 years old

1. Recent Level of education *(Please tick the appropriate box)*:

- Elementary school
- Middle school
- High school
- Undergraduate
- Postgraduate

1. Field of education *(Please tick the appropriate box)*:
   - Related to Information Technology
   - Not related to Information Technology
2. Field of occupation*(Please tick the appropriate box)*:

- Government
- Industry
- Education
- Banking
- Others, please specify : _____________
- Unemployed

1. The smart device you owned*(Please tick the appropriate box, you can choose more than one)*:

- Smartphone (example: iOS-based handphone, Android-based handphone, Windows phone, Blackberry, etc.)
- Smartwatch (example: Apple Smartwatch, Samsung Gear, etc.)
- Smartband/Fitness Tracker/Activity Tracker (example: Fitbit, Mi Band, etc.)
- Smart TV (example: Samsung Smart TV, Sony Smart TV, Google Chromecast-built in Smart TV, etc.)
- Smart speaker(example: Google Home, Amazon Echo, etc.)
- Others
- Do not have any smart device

1. Please specify the brand of smart device you have: (example: iPhone, Apple Smartwatch, Google Home)

Your Answer: _______________________________________________________________

1. **User Perspectives on the Effectiveness of Security Education, Training and Awareness (SETA) Programs**

This section is to find out the perspective of respondents as smart device users on the effectiveness of existing SETA programs. *Please rate the extent to which you disagree/agree with the following statements.*

1. The government has provided training that has a positive impact in helping me as a user to increase my awareness about information security issues

|  | 1 | 2 | 3 | 4 | 5 |  |
| --- | --- | --- | --- | --- | --- | --- |
| Strongly Disagree |  |  |  |  |  | Strongly Agree |

1. The government-run program in educating users about smart device user responsibilities is very effective

|  | 1 | 2 | 3 | 4 | 5 |  |
| --- | --- | --- | --- | --- | --- | --- |
| Strongly Disagree |  |  |  |  |  | Strongly Agree |

1. The government-run program in educating users about the consequences of using smart devices is very effective

|  | 1 | 2 | 3 | 4 | 5 |  |
| --- | --- | --- | --- | --- | --- | --- |
| Strongly Disagree |  |  |  |  |  | Strongly Agree |

1. **User Perspectives on Regulatory Effectiveness**

This section is to find out the perspective of respondents as smart device users on the effectiveness of existing regulations in protecting users. *Please rate the extent to which you disagree/agree with the following statements.*

1. Existing regulations have protected me against the misuse of personal information very effectively.

|  | 1 | 2 | 3 | 4 | 5 |  |
| --- | --- | --- | --- | --- | --- | --- |
| Strongly Disagree |  |  |  |  |  | Strongly Agree |

1. Existing regulations govern how my personal information is collected, used, and protected very effectively.

|  | 1 | 2 | 3 | 4 | 5 |  |
| --- | --- | --- | --- | --- | --- | --- |
| Strongly Disagree |  |  |  |  |  | Strongly Agree |

1. Existing regulations control the use of sanctions for violations or misuse of my personal information very effectively.

|  | 1 | 2 | 3 | 4 | 5 |  |
| --- | --- | --- | --- | --- | --- | --- |
| Strongly Disagree |  |  |  |  |  | Strongly Agree |

1. **User perspectives on the Effectiveness of Smart Device Provide Protection**

This section is to find out your perspectives as a smart device user toward the protection of providers(i.e., hardware providers, operating systems, and applications) to perceived user privacy. *Please rate the extent to which you disagree/agree with the following statements.*

1. Smart device providers have provided a clear and understandable privacy policy statement.

|  | 1 | 2 | 3 | 4 | 5 |  |
| --- | --- | --- | --- | --- | --- | --- |
| Strongly Disagree |  |  |  |  |  | Strongly Agree |

1. Existing privacy policies make me more aware of my rights

|  | 1 | 2 | 3 | 4 | 5 |  |
| --- | --- | --- | --- | --- | --- | --- |
| Strongly Disagree |  |  |  |  |  | Strongly Agree |

1. Smart device providers use reliable technology to protect the security of my personal information

|  | 1 | 2 | 3 | 4 | 5 |  |
| --- | --- | --- | --- | --- | --- | --- |
| Strongly Disagree |  |  |  |  |  | Strongly Agree |

1. Smart device providers give flexibility for me as the user to manage the mechanism of securing personal data.

|  | 1 | 2 | 3 | 4 | 5 |  |
| --- | --- | --- | --- | --- | --- | --- |
| Strongly Disagree |  |  |  |  |  | Strongly Agree |

1. **User Perspectives on Privacy Considerations**

This section is to find out your perspective as the smart device user on privacy issues. *Please rate the extent to which you disagree/agree with the following statements.*

1. I feel disturbed when smart device providers ask for personal information

|  | 1 | 2 | 3 | 4 | 5 |  |
| --- | --- | --- | --- | --- | --- | --- |
| Strongly Disagree |  |  |  |  |  | Strongly Agree |

1. When smart device providers ask me for personal information, sometimes I think first to consider my privacy before giving it.

|  | 1 | 2 | 3 | 4 | 5 |  |
| --- | --- | --- | --- | --- | --- | --- |
| Strongly Disagree |  |  |  |  |  | Strongly Agree |

1. I object to giving out personal information to many parties

|  | 1 | 2 | 3 | 4 | 5 |  |
| --- | --- | --- | --- | --- | --- | --- |
| Strongly Disagree |  |  |  |  |  | Strongly Agree |

1. I am worried that smart device providers collect too much of my personal information

|  | 1 | 2 | 3 | 4 | 5 |  |
| --- | --- | --- | --- | --- | --- | --- |
| Strongly Disagree |  |  |  |  |  | Strongly Agree |

1. Smart device providers should work harder to secure the user’s personal information

|  | 1 | 2 | 3 | 4 | 5 |  |
| --- | --- | --- | --- | --- | --- | --- |
| Strongly Disagree |  |  |  |  |  | Strongly Agree |

1. **User Perspectives of Trust in Technology**

This section is to find out your perspective as a smart device user towards trust in the technology. *Please rate the extent to which you disagree/agree with the following statements.*

1. When asked to share personal information online and I know that information is protected, I feel comfortable with my smart device provider

|  | 1 | 2 | 3 | 4 | 5 |  |
| --- | --- | --- | --- | --- | --- | --- |
| Strongly Disagree |  |  |  |  |  | Strongly Agree |

1. I can count on my smart device provider not to misuse my personal information without my permission

|  | 1 | 2 | 3 | 4 | 5 |  |
| --- | --- | --- | --- | --- | --- | --- |
| Strongly Disagree |  |  |  |  |  | Strongly Agree |

1. I can depend on my smart device provider to comply with all government regulations related to protecting user data

|  | 1 | 2 | 3 | 4 | 5 |  |
| --- | --- | --- | --- | --- | --- | --- |
| Strongly Disagree |  |  |  |  |  | Strongly Agree |

1. **Subjective Norms of Positive Security Behavior**

This section is to find out your perspectives as a smart device user of subjective norms of positive security behavior. *Please rate the extent to which you disagree/agree with the following statements.*

1. Esteemed colleagues for me believe that I must maintain my personal information

|  | 1 | 2 | 3 | 4 | 5 |  |
| --- | --- | --- | --- | --- | --- | --- |
| Strongly Disagree |  |  |  |  |  | Strongly Agree |

1. My family believes that I must be careful about exposing my personal information

|  | 1 | 2 | 3 | 4 | 5 |  |
| --- | --- | --- | --- | --- | --- | --- |
| Strongly Disagree |  |  |  |  |  | Strongly Agree |

1. Influential community leaders for me believe that I must be careful about exposing my personal information

|  | 1 | 2 | 3 | 4 | 5 |  |
| --- | --- | --- | --- | --- | --- | --- |
| Strongly Disagree |  |  |  |  |  | Strongly Agree |

1. **User’s Attitude toward Positive Security Behavior**

This section is to find out your perspectives as a smart device user toward positive security behavior. *Please rate the extent to which you disagree/agree with the following statements.*

1. I believe that implementing security measures on my smart device is a good thing

|  | 1 | 2 | 3 | 4 | 5 |  |
| --- | --- | --- | --- | --- | --- | --- |
| Strongly Disagree |  |  |  |  |  | Strongly Agree |

1. Taking security measures on my smart devices is important

|  | 1 | 2 | 3 | 4 | 5 |  |
| --- | --- | --- | --- | --- | --- | --- |
| Strongly Disagree |  |  |  |  |  | Strongly Agree |

1. **Perceived Behavioral Control toward Positive Security Behavior**

This section is to find out your perspectives as smart device user on controlling behavior toward towards positive security behavior. *Please rate the extent to which you disagree/agree with the following statements.*

1. I have control over the personal information released by smart devices

|  | 1 | 2 | 3 | 4 | 5 |  |
| --- | --- | --- | --- | --- | --- | --- |
| Strongly Disagree |  |  |  |  |  | Strongly Agree |

1. I have control over anyone who can gain access to personal information

|  | 1 | 2 | 3 | 4 | 5 |  |
| --- | --- | --- | --- | --- | --- | --- |
| Strongly Disagree |  |  |  |  |  | Strongly Agree |

1. I have control over how device providers use personal information

|  | 1 | 2 | 3 | 4 | 5 |  |
| --- | --- | --- | --- | --- | --- | --- |
| Strongly Disagree |  |  |  |  |  | Strongly Agree |

1. I am sure I can control my personal information that is given to smart device

|  | 1 | 2 | 3 | 4 | 5 |  |
| --- | --- | --- | --- | --- | --- | --- |
| Strongly Disagree |  |  |  |  |  | Strongly Agree |

1. **User Positive Security Behavior**

This section is to find out your perspectives as a smart device user on the positive security behavior. *Please rate the extent to which you disagree/agree with the following statements.*

1. I believe that reading the privacy policy statement carefully before using a smart device is important.

|  | 1 | 2 | 3 | 4 | 5 |  |
| --- | --- | --- | --- | --- | --- | --- |
| Strongly Disagree |  |  |  |  |  | Strongly Agree |

1. I know where to report if an incident occurs related to the security of my smart device

|  | 1 | 2 | 3 | 4 | 5 |  |
| --- | --- | --- | --- | --- | --- | --- |
| Strongly Disagree |  |  |  |  |  | Strongly Agree |

1. I know of privacy issues related to the use of the smart device that I have

|  | 1 | 2 | 3 | 4 | 5 |  |
| --- | --- | --- | --- | --- | --- | --- |
| Strongly Disagree |  |  |  |  |  | Strongly Agree |

1. I know how to control my personal information given to smart devices

|  | 1 | 2 | 3 | 4 | 5 |  |
| --- | --- | --- | --- | --- | --- | --- |
| Strongly Disagree |  |  |  |  |  | Strongly Agree |

1. I can control the protection of my personal information on all the devices that I have

|  | 1 | 2 | 3 | 4 | 5 |  |
| --- | --- | --- | --- | --- | --- | --- |
| Strongly Disagree |  |  |  |  |  | Strongly Agree |

**Open Question:**

This section explores privacy issues that are most noticed by smart device users. *Please fill in the blank with your answer.*

In your opinion, what are the most important privacy issues to consider when using smart devices?

Your answer: _______________________________________________________________________

Are you willing to be recontacted regarding this research? *(Please tick the appropriate box)*

- Yes, please invite me.
- No, thanks.

**Closing statement**

I appreciate your time and participation in this survey.

Have a beautiful day!

Kind regards,

Kautsarina
